# Clinicopathological features associated to MiRNA-195 expression in patients with breast cancer: Evidence of a potential biomarker

**DOI:** 10.12669/pjms.335.13008

**Published:** 2017

**Authors:** Farkhunda Nadeem, Muhammad Hanif, Akhtar Ahmed, Qamar Jamal, Adnan Khan

**Affiliations:** 1 Dr. Farkhunda Nadeem, Isra University, Hyderabad, Pakistan; 2 Dr. Muhammad Hanif, Karachi Institute of Radiotherapy and Nuclear Medicine, Karachi, Pakistan; 3 Dr. Akhtar Ahmed, Karachi Institute of Radiotherapy and Nuclear Medicine, Karachi, Pakistan; 4 Dr. Qamar Jamal, Ziauddin Medical University, Karachi, Pakistan; 5 Mr. Adnan Khan, Karachi Institute of Radiotherapy and Nuclear Medicine, Karachi, Pakistan

**Keywords:** Breast cancer, Clinicopathological data, miRNA-195

## Abstract

**Background & Objective:**

MiRNAs are a systematic class of small non-coding RNAs with impending role as tumor biomarkers. Our objective was to identify the level of expression of Mir-195 in patients with breast cancer along with its correlation with clinicopathologic features.

**Methods:**

A total of 209 females in which 139 histologically diagnosed breast cancer (BC) cases and 70 healthy controls matched for age, their relative clinical and histopathological findings were recorded from their laboratory reports and hospital record of the patients. Plasma was used for extraction of total RNA and cDNA was prepared by using both miR-195 stem loop RT primers and gene specific antisense primers while U6 IT was used as control. Quantitative real-time PCR (qRT-PCR) for miR-195 expression status was performed and amplification (down regulation) was recorded.

**Results:**

Of 139 samples the expression of miR-195 was down regulated in 72.6% cases and the remaining 27.3% cases behaved same as 70 healthy or normal controls. Significant correlation of low miR-195 expression with higher differentiation grade, lymph node metastasis and clinical stage was found.

**Conclusion:**

Significant correlation between miR-195 expression and some clinicopathological features were recognized. MiR-195 could be used as potential non-invasive, molecular biomarker for early detection of breast cancer.

## INTRODUCTION

Breast cancer is the most frequently reported and diagnosed cancer in women worldwide and is principal cause of cancer related death among females.^1^ In Pakistan, the Age-standardized rate/100,000 is highest in the world and probability of having breast cancer is one out of nine women, and is likely for 40,000 deaths every year out of the 90,000 cases detected.^2^ Accordingly Pakistani women have the highest risk of breast cancer among all Asian populations which is already reported by several authors.^3^ The situation become worst when patients with breast cancer have metastasis, a situation which considerably elevates the tumor load frequently resulting in serious result.^4^ Timely diagnosis and accordingly management of breast cancer can improve the five year survival rate as well as the quality of life.^5^

MiRNAs are small, 18–24 nucleotide long specific class of non-coding RNAs that are preserved across species and contribute in gene silencing at post-transcriptional level.^6^ Current molecular diagnostics of breast cancer and the potential incorporation of microRNAA lot of studies have reported that miRNAs play important roles in many human biological and pathological pathways such as growth, apoptosis, development and tumorigenesis.^7^ The role of tumor- associated miRNAs is now established as tumor suppressors or oncogenic miRNAs.^8^

These findings offer experimental support for using miR-155 as a therapeutic intervention for the treatment of breast carcinoma.^9^ MiR-146 a/b is found to acts as a tumor suppressor gene and can also be useful as a biomarker for identification of breast cancer. It is reported to be dysregulated in different pathways leading to the development of breast cancer.^10^ Another miRNA miR- 485 is found to be down-regulated in breast cancer indicating its tumor suppressor role in BC. It’s down regulation play an important role in development and progression of breast cancer and may serve as potential biomarker^11^. Similarly many miRNAs can act as oncogenic in breast cancer. The constancy of miRs and their occurrence in blood and in body fluids, such as serum and plasma, open up the likelihood of using miRNA as biomarkers for detection of cancers including breast cancer.^12^ We are interested to focus on miR-195, which has been reported to be downregulated in a wide range of malignant tumors, including hepatocellular carcinoma^13^, colorectal cancer^14^, gastric cancer^15^ and breast cancer.^16^

We carried out Real Time PCR assays initially on tissues followed by on plasma to detect the expression of miR-195 in BC patients and controls. Moreover, the correlations of miR-195 expression with clinicopathological features of BC patients were statistically analyzed. Our data showed that miR-195 was significantly down regulated in plasma of BC patient and could be served as a potential non-invasive molecular biomarker for the early detection of BC.

## METHODS

### a) Patients and blood samples

The study and the data collection were carried out at Liaquat University Hospital (LUH) Jamshoro, Isra University Hospital Hyderabad and Karachi Institute of Radiotherapy and Nuclear Medicine (KIRAN), Karachi. The study lasted for a period of one year. The cases were selected randomly for age, parity and social class. Patients having any other cancer beside breast cancer were not included in the study. Patient consent was taken on proforma from all study participants. Plasma was separated from each EDTA bottle after two hours in 2 ml Eppendorf tubes and stored at – 40°C and processed for the extraction of RNA. Patient’s demographic data, history, biopsy and grading of the tumors were documented from the laboratory reports and clinical data were recorded from the hospital HIMS record.

### b) Extraction of RNA

Total RNA (including MiRNAs) from all the plasma samples was extracted by TRIZOL LS method according to the protocol provided by the suppliers (invitrogen).

### c) Reverse transcriptase reactions

The cDNA was synthesized from total RNA as per cDNA kit instructions of Thermo Scientific. Total RNA from each sample was reverse transcribed into cDNA with an oligo (dT) primer. PCR primers already reported^14^ were synthesized by IDT and each sample were analyzed for each specific gene with Sybr Green I Master mix on the Bio-Rad CFX system according to manufacturer protocols (Bio-Rad USA). The average *C* t value of the endogenous control (GAPDH) for every sample was subtracted from the *C* t value for each target gene, resulting in the Δ*C* t value. MiRNA expression levels were calculated using ΔΔ*C* t method.^17^

### d) MiRNA-specific real time PCR (miR-RT-qPCR)

Total RNA was poly(A) tailed using poly(A) polymerase (NEB) at 37°C for one hour, then terminated by heating at 65°C for 20 min. Poly(A)-tailed RNA (1.2 μg) was then reverse-transcribed into first-strand cDNA using reverse transcriptase with miRNA specific stem-looped RT primer.^14^

### e) Quantitative reverse transcription (qRT)-PCR

Thermo Scientific Maxima SYBR green/ROX qPCR Master Mix (2x) was used for PCR. Real Time PCR amplification in the form of CT (Threshold cycle) was noted and quantification was done by ΔΔCt cycle threshold method after normalization to that of U6 (used as internal control).

### f) Statistical analysis

Data analysis was done on SPSS version 21.0 for windows release (IBM, incorporation, USA). Variables were analyzed using students t-test and Chi-square test. A *P* value of ≤ 0.05 was defined as significant.

## RESULTS

### 1. Demography of patients

All 139 females with biopsy confirmed BC was included in this study. Mean age was 41.3±5.13 years. Most of the BC patients 99 (71.22%) belonged to 5^th^ decade of life, after that 6^th^ decade noted in 31 (22.30%) of cases. Only two (1.43%) patients were in 3^rd^ decade (*p*=0.001). Marital status (married to unmarried) ratio was 33.75:1, only four patients (2.9%) were unmarried while 135 (97.1%) were married. Out of 139 cases 38 (27.3%) were post-menopausal and 101 (72.66%) were pre-menopausal female (Table-I).

**Table-I:** Demography of study population (n=139).

		*No. of Pt.*	*%*	**p*-value*
1. Age Distribution				
a	25-29.9 years	2	1.4	
b	30-39.9 years	7	5.0	0.001
c	40-49.9 years	99	78.4	
d	>50 years	31	15.1
e	Marital Status			
f	Married	135	97.1	0.001
g	Unmarried	4	2.9	
2. Menstrual History				
a	Post-menopause	38	27.3	0.02
b	Pre-menopause	101	72.66	
c	Regular menstrual cycle	71	70.2	
d	Irregular menstrual cycle	30	29.70	
3. Familial History				
a	H/O BC in 1st Degree relatives	21	(15%)	0.001
b	H/O Breast feeding	139	(100%)	
c	H/O taking Hormonal Pills	52	(37.4%)	0.001
d	H/O Contraception	23	(16.5%)	

### 2. Clinical examination

Eighty two (58.9%) cases had cancerous lumps in their right breast followed by 57 (41%) in left breast, bilateral breast lumps were not observed in present study. Only 4.3% cases had more than one lump and majority of cases (95.6% of cases) had one lump. Lump size of more than five cm was noted in 91.3% of cases, while small sized lumps i.e., less than two cm were found only in 0.71% of cases (Table-II).

**Table-II:** Clinical examination of BC patients (n=139).

		*No. of Pt.*	*%*	**p*-value*
1. Laterality of breast Lump				
a	Bilateral breast	0	0	
b	Right breast	82	58.9	0.001
c	Left breast	57	41	
2. No of breast Lump				
a	One lump	133	95.6	0.001
b	>1 lump	6	4.3	
3. Size of breast Lump				
d	< 2 cm lump	1	0.71	
e	> 2 - < 5 cm lump	11	7.91	0.001
f	> 5cm lump	127	91.3	
4. Consistency of breast Lump				
a	Firm	74	53.2	
b	Hard	65	46.7	0.001
5. Margins of breast Lump				
a	Regular	69	49.6	0.001
b	Irregular	70	50.3	
6. Involvement of Lymph Node				
a	Not involved	50	35.97	0.056
b	Involved	89	64.01	
c	1 lymph node	50	56.17	
d	>1 lymph node	39	43.82	
e	Attachment to	Yes	No	
f	Skin	63(45.3%)	76(54.6%)	0.07
g	Underlying structure	61(43.8%)	78(56.1%)	

### 3. Histological examination

Invasive Ductal carcinoma was in 138 (99.2%) of patients while invasive lobular carcinoma was observed only in one case (carcinoma in situ was not observed in our study (Table-III).

**Table-III:** Histological examination and Clinical staging (n=139).

		*No. of Pt.*	*%*	**p*-value*
1. Histological sub-types of Breast Cancer				
a	Carcinoma in situ	0	0
b	Invasive carcinoma	139	100	0.001
c	Invasive Ductal carcinoma	138	99.2	
d	Invasive lobular carcinoma	1	0.71	
2. Grade of tumor				
a	Grade I.	27	19.4	
b	Grade II.	92	66.1	0.001
c	Grade III.	20	14.3	
3. Other findings of breast cancer				
a	Inflammatory response	111	28
b	Blood vessel Involved	1	138	0.001
c	Angiogenesis	9	130
4. Clinical staging of BC (AJCC) (n=139)				
a	Stage 0	0	0
b	Stage I.	21	15.10	0.001
c	Stage II.	39	28.05	
d	Stage III.	77	55.39	
e	Stage IV.	2	1.43	

Majority of cases 92 (66.18%) consist of Grade II. Grade I and III were noted in 27 (19.4%) and 20 (14.3%) cases respectively (*p*=0.001). Clinical staging was done according to AJCC. Majority of breast cancer were found in stage III i.e. 77 (55.39%), followed by Stage II, I and IV respectively (*p*=0.001) (Table-III).

### 4. Down regulation of miR-195

Out of 139 breast cancer cases, 38 (27.3%) showed high expression and 101(72.6%) showed low expression. On the contrary, controls (n=70) showed higher and low expression in 59(84.2%) and 11(15.7%) of cases respectively (Table-IV).

**Table-IV:** Expression of miR-195 in Breast cancer and controls (n=139).

		*Breast cancer (n=139)*	*Controls (n=70)*	**p*-value*
a	Higher	38	59	0.001
b	Lower	101	11	0.001
Expression of miR-195 in different age categories (n=139)				
1	Age	*High Expression (n=38)*	*Low Expression (n=101)*	*p*-value
a	25-29.9 years	0	2	0.001
b	30-39.9 year	3	4	0.001
c	40-49.9 years	27	72	0.002
d	>50 year	8	23	0.03
2. History of hormonal pills				
a	Yes	13	39	0.001
b	No	25	62	0.001
3. History of breast cancer in first degree relatives				
a	Yes	7	14	0.08
b	No	31	87	0.09
4. Size of breast lump				
a	< 2 cm	0	1	0.06
b	2-5 cm	3	8	0.071
c	>5 cm	35	92	0.05
Association of miR-195 expression with Lymph node involvement (n=139)				
		*High Expression (n=38)*	*Low Expression (n=101)*	*p*-value
a	Not involved	13	37	0.001
b	Involved	25	64	0.001
c	1 lymph node	11	39	0.06
d	>1 lymph node	14	25	0.053
	Association of miR-195 expression with histological grades (n=139)			
		*High Expression (n=38)*	*Low Expression (n=101)*	*p*-value
a	Grade I	10	17	0.001
b	Grade II	23	69	0.001
c	Grade III	5	15	0.001
Association of miR-195 expression to clinical stage (n=139)				
		*High Expression (n=38)*	*Low Expression (n=101)*	*p*-value
a	Stage I	4 (2.8%)	17 (12.2%)	0.003
b	Stage II	14(10.0%)	25 (17.9%)	0.002
c	Stage III	20(14.3%)	57 (41%)	0.001
d	Stage IV	0(%)	2(1.4%)	0.001

### 5. Association of clinicopathological features with miR-195 expression

Out of 89 BC patients who were presented with axillary lymph node involvement, 71.9% showed downregulation of miR-195 with highly significant *p*-value i.e., 0.001. In contrast out of 50 cases with no axillary lymph nodes, 74% of the cases showed downregulation of miR-195 with same highly significant *p*-value i.e., 0.001 (Table-IV).

Out of 19.4% of grade I, 66.1% of grade II and 14.38% of grade III cases of BC, 62.96% (*p*=0.001), 75% (*p*=0.001) and 75% (*p*=0.001) of the cases revealed down regulation of MiR-195 respectively (Table-IV).

Out of 15.1% of stage I, 28.05% of stage II, 55.39% of stage III and 1.43% of stage IV cases of BC, 80.95%, 64.1%, 74% and 100% (*p*=0.001) of the cases showed down regulation of MiR-195 (Table-IV).

## DISCUSSION

Worldwide breast cancer (BC) has reached the top most position amongst the cancers in women.^18^ Early detection of cancer is vital for saving the lives of patients. This could be achieved by screening programs, self examination, and awareness about it. Studies have revealed that regardless of the high incidence and death ratio related to BC, early detection is still not possible because of lack of knowledge and wakefulness about it even among the educated females like students of medical and non-medical universities.^5^

MiRNAs signify a new biological entity with impending role as tumor biomarkers, which can helpful in diagnosis.^19^ Several studies about role of MiRNA and cancers highlighted the possible function of miRNAs as molecular biomarkers in many human cancers as well as BC.^20^

A large number of miRNA show dysregulation in BC. These miRNAs are useful in diagnosis of cancers as well as in their prognosis as reported by Tang and collogues^21^ that the increase in expression of miR-27 in breast cancer patients was associated with overall poor survival, signifying that miR-27a could be an important marker of development of carcinoma of breast.^22^

Reason of selecting MiR-195 for this study was that miR-195 is highly conserved small non coding RNAs clustered at the same Chromosomal region 17p13.1 which play key role as tumor suppressors in BC.^23^

In this study we have reported expression of MiR-195 in BC which has been found deregulated in a large number of malignant tumors, including hepatocellular carcinoma^13^, colorectal cancer^14^, gastric cancer^15^ and breast cancer.^16^ The results of the present study showed that the relative expression level of miR-195 in BC was considerably lower than in non-cancerous plasma samples. These results are supported by the findings of Dan Li et al.^16^ who reported that expression levels of miR-195 and miR-497 are inversely correlated with BC. Such expression pattern may well identify malignant tumors from normal or benign tumors.^15^

Analysis of association of miR-195 with clinicopathological features showed that status of miR-195 expression in plasma samples of BC patients was significantly correlated with tissue differentiation grade, lymph node metastasis and clinical stage of BC patients. Zhao et al.^24^ has observed two fold reductions in the status of miR-195 level in BC patients as compared to healthy control group. He has reported the usefulness of MiRNAs for early detection of tumor but has not found any significant relationship of it with TNM staging and clinicopathological parameters. We have highlighted the importance of expression of MiR-195 in BC patients with weak differentiation grade, higher occurrence of lymph node metastasis and advanced clinical stage, suggesting that down regulation of miR-195 played an important role in BC development. We have also found that although low miR-195 expression is significantly correlated with advanced clinical stage but its expression start decreasing from the stage II and even in stage I. These results highlights another fact that level of MiR-195 is significantly down regulated in all grades and stages of BC, even in lymph node negative patients with a highly significant *p*-value in all of these parameters. This property makes this miRNA a potential marker for diagnosis of BC in early stages.

## CONCLUSION

It was found that there is noticeably significant relationship of low miR-195 expression with higher differentiation grade and lymph node metastasis and clinical stage with significant p-value. Moreover, there were significant association between miR-195 expression and some clinicopathological features. Majority of BC patients from SINDH present in 5^th^ decade of life and most of these patients were married, pre-menopausal signifying the occurrence of BC in young age group. Breast cancer in first degree relative was present in small number of patients. All the BC females have breast fed their children. Very small numbers of BC patients have taken exogenous hormones in the form of pills or have had any mode of contraception. All the patients were having unilateral breast cancer. The main bulk of the BC patients presented with one lump with the size more than 5 cm. Majority of the patients had axillary lymph node involvement at the time of presentation; a fact highlighting late presentation and diagnosis of BC. All the cases were invasive ductal carcinoma at grade II differentiation and in stage III at the time of presentation.

### Authors’ Contributions

**FN, MH** conceived, designed and did statistical analysis & write up with editing of manuscript.

**AK** did data collection and bench working.

**AA, QJ** review work and final approval of manuscript.
